# Iron Overload in Patients Undergoing Hematopoietic Stem Cell Transplantation

**DOI:** 10.1155/2010/345756

**Published:** 2010-09-08

**Authors:** Vinod Pullarkat

**Affiliations:** Department of Hematology and Hematopoietic Cell Transplantation, City of Hope Medical Center, 150 East Duarte Road, Duarte, CA 91010, USA

## Abstract

Recipients of hematopoietic stem cell transplantation (HSCT) frequently have iron overload resulting from chronic transfusion therapy for anemia. In some cases, for example, in patients with myelodysplastic syndromes and thalassemia, this can be further exacerbated by increased absorption of iron from the gut as a result of ineffective erythropoiesis. Accumulating evidence has established the negative impact of elevated pretransplantation serum ferritin, a surrogate marker of iron overload, on overall survival and nonrelapse mortality after HSCT. Complications of HSCT associated with iron overload include increased bacterial and fungal infections as well as sinusoidal obstruction syndrome and possibly other regimen-related toxicities. Based on current evidence, particular attention should be paid to prevention and management of iron overload in allogeneic HSCT candidates, especially in patients with thalassemia and myelodysplastic syndromes. The pathophysiology of iron overload in the HSCT patient and optimum strategies to deal with iron overload during and after HSCT require further study.

## 1. Introduction

Hematopoietic stem cell transplantation (HSCT) is increasingly used as curative therapy for a variety of disorders of the hematopoietic and immune systems. Although considerable advances in transplantation practice have resulted in greater overall survival rates, transplant-related mortality is a major hurdle to improving HSCT outcome, especially in older patients and those who are transplanted in the later stages of their hematologic disorder. Transplantation outcomes can vary greatly among diseases and are impacted by a range of complications such as infections, graft-versus-host disease (GVHD), and toxicities related to the conditioning regimen, including hepatic sinusoidal obstruction syndrome (SOS) [1]. Iron overload is a common problem in red cell transfusion-dependent patients who undergo HSCT. The strongest evidence for the adverse impact of iron overload on HSCT outcome comes from the thalassemia literature [2]. In recent years, the role of iron overload as a risk factor in nonthalassemic HSCT transplant recipients has also been widely investigated due to its potential impact on patient morbidity and mortality. This paper discusses the current literature on iron overload as it pertains to the HSCT patient and outlines areas for future research.

## 2. Iron Overload in the HSCT Patient

**Figure 1:**
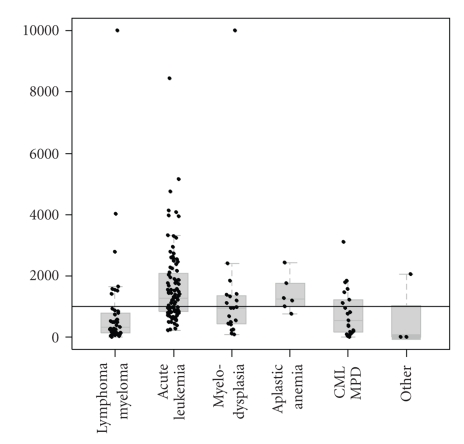
Iron overload in patients undergoing HSCT [8]. Dots represent individual patients, thick grey lines show median values, and the boxes indicate interquartile range. Horizontal black line represents a serum ferritin level of 1000 ng/mL. CML: chronic myeloid leukemia; MPD: myeloproliferative disorder [8].

Iron overload, as measured by pre-transplantation serum ferritin, is common in recipients of HSCT, particularly in those with hemoglobinopathies, acute leukemia, and myelodysplastic syndromes (MDSs) (Figure 1). Red blood cell transfusion therapy as supportive care for chronic anemia is the principal cause of iron overload in such patients. Patients may also require further transfusion therapy following conditioning and prior to engraftment. As each unit of transfused packed red cells contains approximately 200–250 mg of iron, patients who are administered regular transfusions can receive a daily iron excess of up to 0.5 mg/kg [3]. With no physiologic mechanism for clearing excess iron taken in as a result of transfusions, iron accumulation is an inevitable sequel, and patients can become iron overloaded after as few as 10–20 transfusions [4]. In some patients, such as those with MDS and thalassemia, the underlying conditions for which they are being treated may cause them to have excessive iron absorption from the gut as a consequence of ineffective erythropoiesis. This effect is mediated by erythroid regulators of iron metabolism, which suppress hepcidin and, in turn, result in increased iron absorption from the duodenum.

## 3. Pathophysiology of Iron Overload

As iron can readily donate and accept electrons, interconverting between ferrous (Fe^2+^) and ferric (Fe^3+^) forms, it is an important component of various cytochromes and is required for the functioning of a number of enzymes. However, this property also makes iron highly toxic, being able to catalyze the conversion of hydrogen peroxide into free-hydroxyl radical ions that can damage cellular membranes, proteins, and DNA [5]. Under normal conditions, the potential toxicity of plasma iron is eliminated by sequestration into complexes with transferrin, the major plasma iron-binding protein. However, the presence of excess body iron can quickly saturate the available transferrin and result in the appearance of nontransferrin-bound iron (NTBI). The pathologically relevant component of NTBI is labile plasma iron (LPI), which encompasses organ-penetrating forms of iron that are redox active and directly chelatable [6]. Generation of LPI leads to unregulated iron uptake and subsequent intracellular storage either within ferritin molecules or as hemosiderin, an iron storage complex. When the sequestering capacity of iron-binding proteins is exceeded, excessive labile iron pools develop, which are the mediators of organ toxicity in iron-overloaded patients [7]. In the absence of treatment the toxic effects of the stored iron will result in ongoing tissue damage and ultimately organ dysfunction and failure [4]. Therefore, the adverse consequences of iron overload can arise from the elevation of NTBI and LPI in plasma, as well as due to organ damage mediated by the accumulation of tissue iron in target organs. The sources of elevated NTBI in HSCT patients are summarized in Table 1. 

Iron from red cell transfusions initially accumulates bound to ferritin in the macrophages of tissues such as liver, spleen, and bone marrow. Iron export into plasma from iron-loaded macrophages as well as duodenal enterocytes (in the case dietary iron absorption) occurs via the iron export protein ferroportin located on the membrane of these cells [9]. Ferroportin expression in turn is negatively regulated by hepatic production of hepcidin, a key hormone that regulates iron metabolism by inducing the internalization and degradation of ferroportin [10]. Serum hepcidin-25 levels have been shown to negatively correlate with the degree of erythropoiesis in the HSCT setting [11]. Thus, the degree of erythropoietic activity after HSCT appears to be the major regulator of hepcidin level which in turn may determine plasma iron levels in the HSCT setting.

**Figure 2:**
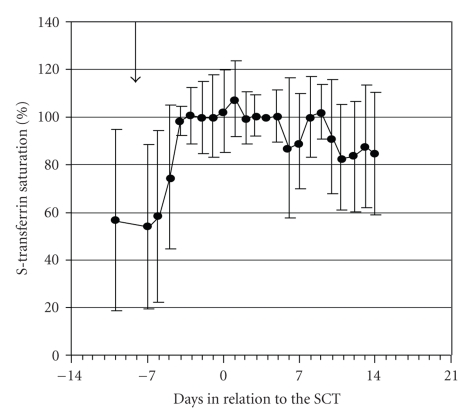
Mean ± SD serum level of the calculated transferrin saturation in 10 allogenic SCT patients during the peritransplantation period. Arrow indicates onset of the conditioning regimen [12].

The conditioning regimen itself can also contribute to the increase in NTBI levels, partly due to inhibition of erythropoiesis, the main route of iron utilization. In one study, NTBI peaked as early as 4 days prior to transplantation, and was detectable for 6–18 days in all patients [12] (Figure 2). Other studies have shown similar results [13, 14]. In addition, stored iron can be released from the liver as a result of tissue injury that can occur during conditioning [12, 15, 16]. Transferrin levels can drop as an acute phase reaction, and transferrin saturation can increase during chemoradiotherapy, often reaching indexes of more than 80% and leading to increased levels of NTBI [12, 13, 16]. Conditions in the peritransplantation period can therefore result in elevated NTBI and LPI levels in the plasma, which persist at least until engraftment. 

Normally, endogenous antioxidants also play a role in scavenging free radicals and preventing cell damage [17]. However, in patients undergoing HSCT, chemotherapy or radiotherapy-based conditioning regimens can result in a prooxidant status, as indicated by a reduced total radical antioxidant parameter of plasma (TRAP), a measure of the overall capacity of human plasma to inhibit free radical-induced lipid peroxidation [14, 18]. In one study, assessment of the antioxidant status before and after HSCT showed a breakdown in plasma antioxidant defense and an inverse correlation between levels of NTBI and TRAP. Recent data have also demonstrated a prooxidant state in patients conditioned with chemoradiotherapy, indicated by significant increases in malondialdehyde (an indicator of oxidative stress and lipid peroxidation), glutathione peroxidase, and super oxide dismutase [19]. Decreased levels of other endogenous antioxidants such as *α*-tocopherol and *β*-carotene have also been noted [14, 20]. The disturbance of pro-oxidative/antioxidative balance in the plasma of patients undergoing HSCT may augment the toxicity of LPI and suggests that the adminstration of antioxidants, such as N-acetylcysteine or glutamine (glutathione precursor), may, therefore, be beneficial [14].

In addition to performing a vital role in the human body, iron is an important element for the growth of pathogenic microorganisms [21, 22]. High plasma iron levels can therefore not only promote microbial growth but can also directly increase susceptibility to infection by inhibiting the function of the immune system. High intracellular iron levels have been shown to result in the direct impairment of innate and acquired immune responses [21].

## 4. Impact of High Pretransplantation Serum Ferritin Levels on Survival and Complications

The adverse impact of iron overload on HSCT outcome was first demonstrated in thalassemia patients. In fact, the degree of iron overload and adequacy of chelation is used for prognostic classification of thalassemia patients undergoing allogeneic HSCT. Multivariate analysis has identified three iron-related factors associated with a significantly reduced probability of survival: hepatomegaly, hepatic portal fibrosis, and inadequate iron chelation [23]. Patients with none of these pre-HSCT risk factors are considered low risk (Class 1), those with one or two are moderate risk (Class 2), and those with all three risk factors are high risk (Class 3). While the probability of long-term survival after HSCT in Class 1 patients may be more than 90%, survival can be anticipated in only around 50% of high risk, Class 3, patients [2, 23].

**Figure 3:**
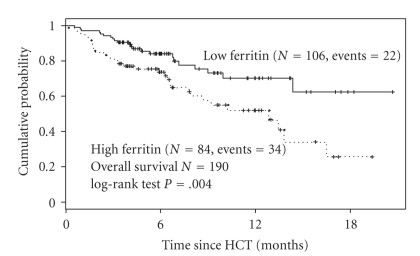
Prognostic impact of elevated pre-transplantation serum ferritin in patients undergoing myeloablative stem-cell transplantation (*N* = 190). Dashed line represents patients with serum ferritin over 1000 ng/ml. Adapted from [8].

In recent years, a number of studies have investigated the effect of high pre-transplantation serum ferritin levels on survival after HSCT in nonthalassemic patients (Table 2). Although most of the studies are retrospective, the results are unequivocal; the increased incidence of complications associated with high iron load results in reduced overall survival after HSCT particularly in patients with MDS and acute leukemia (Figure 3) [8, 24–31]. This is true for both full- and reduced-intensity conditioning regimens for HSCT suggesting that reduction in conditioning intensity alone may not be adequate to minimize the deleterious effects of iron overload on survival. 

Conditioning regimens, cytopenias, and the use of immunosuppressive agents can result in severely compromised immunity. As a consequence, infection accounts for much of the treatment-related mortality observed in HSCT patients. A recent prospective evaluation of 190 HSCT patients has confirmed that serum ferritin values of ≥1000 ng/mL are associated with a significant increase in the incidence of blood stream infections [8]. In this study, patients with pretransplantation serum ferritin above 1000 ng/ml had a twofold increased risk of developing blood stream infections compared to those whose level was below 1000 ng/ml [8]. Increased NTBI has been associated with an increased risk of infection in patients with acute leukemia and those who undergo myeloablative chemotherapy [32–34]. Elevated NTBI in HSCT patients has been shown to support growth of *Staphylococcus epidermidis* [35]. Retrospective studies assessing pre-transplantation bone marrow iron stores show that increased iron stores are a significant risk factor for severe infection, primarily invasive aspergillosis [36, 37]. These data have been supported by assessment of serum ferritin and liver iron concentration (LIC), where elevated iron load is seen to be associated with higher infection levels, particularly in patients who died after HSCT [28, 38, 39]. 

GVHD remains a major cause of morbidity and mortality after allogeneic HSCT [40]. While elevated pre-HSCT serum ferritin levels have been associated with an increased incidence of acute GVHD in some studies [8, 26], this has not been a consistent finding, with others showing no effect, or a decrease in this event [24, 31]. Indeed, a reduction in GVHD would be consistent with the known immunosuppressive effects of iron, but the equivocal findings to date indicate the need for further investigation in prospective studies to determine the association of iron overload and GVHD.

Another contributor to transplant-related mortality is SOS, which occurs primarily as a result of endothelial and hepatocyte damage due to conditioning regimen and is associated with high morbidity and mortality [41–45]. The impact of high iron levels on SOS has been investigated, establishing serum ferritin levels of >1000 ng/mL in the pre-transplant period as a key risk factor for the subsequent development of SOS [46–48]. Hepatic endothelial damage induced by chemotherapy and potentiated by elevated LPI after conditioning can be postulated as a possible mechanism mediating the increased risk of SOS in iron-overloaded patients. 

**Figure 4:**
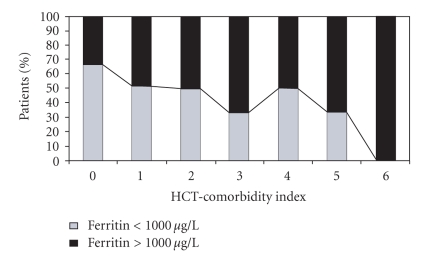
Association of pre-transplantation serum ferritin levels and morbidity [26]. Comorbidity index: 0 = low risk, 1 to 2 = intermediate risk, 3 or more = high risk.

In summary, these data demonstrate that elevated pre-transplantation serum ferritin levels have a significant impact on posttransplant complications and survival. Indeed, pre-HSCT serum ferritin level has recently been included in a prognostic score for patients with MDS or acute leukemia undergoing allogeneic transplantation [49]. The score focuses on five variables: age, disease, stage at transplantation, cytogenetics, and pre-transplant serum ferritin (< or >2500 ng/mL). It has been demonstrated that pre-transplantation comorbidities, including dysfunction of various organs (as categorized by the HSCT-comorbidity index), have a profound impact on transplantation outcome [50]. Subsequent assessment of whether serum ferritin levels correlate with the HSCT-comorbidity index has shown that iron overload is associated with a significantly higher HSCT-comorbidity index (Figure 4) [26].

## 5. Iron Overload in the Post-HSCT Period

As data begin to accumulate, it is apparent that iron overload can persist after HSCT, potentially for many years [51–53]. A recent analysis of data from 77 allogeneic HSCT patients demonstrated that the elevated serum ferritin levels seen in the pre-transplantation period increased further during the posttransplant investigation period of 90 days [54]. In addition, elevated serum ferritin levels have been observed in patients who were independent of red cell transfusions for several years after HSCT [55, 56]. In pediatric patients, iron overload can decrease over time as a result of utilization of storage iron for growth. However, a study of patients who underwent HSCT for thalassemia demonstrated that although a decrease in serum ferritin occurred in all three risk groups (assigned prior to HSCT based on iron overload), ferritin levels normalized only in the low risk groups who were younger and had least iron overload at time of HSCT. This shows that utilization of iron for growth alone cannot normalize iron stores in moderate-to-severely iron-overloaded pediatric patients [57]. In a study of adult patients who had undergone HSCT for acute myeloid leukemia, there was a drop in serum ferritin with time but this decline was not statistically significant [55].

The effects of persistent iron overload on the long-term morbidity of HSCT recipients (particularly as it relates to late organ dysfunction) have not been investigated. These late effects may differ between thalassemic and nonthalassemic patients and may be related to the distribution of tissue iron (parenchymal *versus* macrophage) in different disease states. Such studies will require long-term patient followup as well as use of sensitive measurements for the evaluation of cardiac, hepatic and endocrine dysfunction. Iron overload is known to contribute to the etiology of liver dysfunction chiefly manifesting as elevated transaminases, a common chronic complication occurring in 50%–72% of patients [62, 63]. Iron overload therefore can mimic exacerbation of hepatic GVHD following HSCT, leading to unnecessary continuation or intensification of immunosuppressive therapy [64]. In a study assessing the role of liver biopsy in evaluating the cause of elevated transaminases in post-HSCT patients, 33% of biopsies had evidence of iron overload and no other pathologic findings [63]. Normalization in liver enzymes has been demonstrated with phlebotomy and iron chelation therapy [64, 65]. The role of persistent iron overload in infections that occur late after HSCT, particularly in patients with chronic GVHD requires further investigation.

## 6. Practical Patient Management

Although no data from controlled trials that document a survival impact of managing iron overload in MDS patients prior to undergoing HSCT are currently available, recent MDS guidelines highlight the need for management of iron load in HSCT candidates. The rationale for these recommendations is the particularly strong adverse survival impact of pre-HSCT iron overload in these patients. These guidelines are summarized in Table 3 [58–61].

### 6.1. Assessing Iron Load in the HSCT Patient

Ferritin is a cellular iron storage protein that maintains iron in a soluble and nontoxic form. Under normal conditions, ferritin levels in the serum are low, but steadily increase in conditions of iron overload. Therefore, assessment of serum ferritin levels serves as a simple and widely used surrogate marker for body iron load. Serum ferritin levels are, however, subject to natural fluctuation and can also be greatly affected by a range of conditions that are particularly relevant in the HSCT patient. These include inflammation, liver damage, infection, and GVHD, all of which can result in elevated serum ferritin levels and, therefore, potential overestimation of iron load. Serial serum ferritin measurements can compensate for potential fluctuations to some extent and should be performed to establish a picture of iron overload over time. 

As 90% of excess iron is deposited in the liver, assessment of LIC provides an accurate measure of whole-body iron levels [66]. Measurement of LIC by biopsy is the validated reference standard but increasingly noninvasive magnetic resonance imaging (MRI) techniques are replacing this approach as the technology becomes more widely available [67]. An additional advantage of MRI is its ability to measure cardiac iron, which does not correlate with serum ferritin or hepatic iron. Cardiac iron deposition, although uncommon in nonthalassemic patients can occur in certain patients with MDS [68–70]. As with evaluation of serum ferritin, thresholds for LIC levels have been determined primarily from thalassemia populations, indicating that LIC levels of >7 mg Fe/g dry weight present an increased risk of complications [66, 71–73]. Threshold levels specific to recipients of HSCT have not been investigated, but could differ significantly from other settings and require further investigation. 

A significant correlation between serum ferritin and LIC has been established in regularly transfused patients with thalassemia major [71, 74]. However, in patients with thalassemia intermedia assessment of serum ferritin levels has been shown to significantly underestimate body iron load [75]. As serum ferritin predominantly reflects macrophage iron, such may be the case with certain patients with MDS who have ineffective erythropoiesis leading to increased iron absorption from the gut and increased parenchymal iron. Disease-specific considerations and acute complications are likely to present a complex environment in which to determine the relationships between serum ferritin, LIC, and parenchymal iron in a particular HSCT patient. Studies have determined the correlation between LIC assessed by MRI and serum ferritin levels in long-term survivors of HSCT. In one study of 65 patients who had survived a median of 8.8 years after HSCT, there was a good correlation between LIC and number of red blood cell transfusions (*r* = .84) but only a moderate correlation between LIC and serum ferritin (*r* = .55) [76]. Two other studies that have examined this issue showed only a modest correlation (*ρ* = .47) or no correlation at all between serum ferritin and LIC measured by MRI [77, 78]. Thus, serum ferritin may have limited usefulness as a marker of iron overload in survivors of HSCT and this may be due to acute inflammatory states or hepatic inflammation in many of these patients.

### 6.2. Management of Iron Overload in the HSCT Patient

#### 6.2.1. Timing of Intervention

A key factor in dealing with iron overload in the HSCT recipient is the timing of intervention with relation to HSCT. This depends on whether the major goal is to reduce tissue iron stores or to temporarily lower NTBI and LPI in the period from conditioning to engraftment. The three possible opportunities to intervene are the following.

1. *Prior to HSCT (before initiation of conditioning):* since HSCT often has to be performed quickly in patients with conditions, such as acute leukemia, an opportunity to achieve negative iron balance in transfusion-dependent patients prior to HSCT may be possible only in certain settings, for example intermediate risk MDS where some delay in HSCT is acceptable. Since the impact of serum ferritin on overall survival persists when examined as a continuous variable, it appears that patients would benefit from undergoing HSCT at the lowest possible body iron burden. Randomized prospective studies of immediate HSCT versus HSCT after adequate chelation are feasible in MDS and will be required to determine the role of chelation after a decision to proceed to HSCT has been made.
2. *Immediate peritransplant period (from start of conditioning until engraftment)*: as NTBI levels increase immediately after initiating conditioning and remain elevated at least until engraftment, iron chelation in this HSCT phase could limit the effects of high NTBI levels and offer potential benefits in terms of reduced risk of infection and SOS without lowering total body iron. Prospective studies are needed to demonstrate the benefit of this strategy. The ideal chelator for this phase would be one that does not release bound iron to microbes (unlike deferoxamine, Desferal), but which can be administered intravenously (unlike deferiprone (Ferriprox) and deferasirox (Exjade)). Moreover, chelators considered for use during this phase of HSCT should not have deleterious effects on other HSCT outcomes like engraftment and immune reconstitution or major interactions with immunosuppressive agents. Novel iron-binding agents such as apotransferrin have potential for use during this period but require further evaluation [35].
3. *Late post-HSCT period (after Day +100)*: in long-term adult survivors of HSCT, excess body iron can persist and is generally poorly managed. A comprehensive strategy considering patient diagnosis, comorbidities, and tissue iron distribution appears to be necessary to guide proper management. Awareness of iron overload and effective management in this period has potential to improve long-term patient outcome. Practical treatment options in this phase include phlebotomy or an oral chelator like deferasirox as discussed in detail later.

### 6.3. Therapeutic Options

#### 6.3.1. Phlebotomy

Phlebotomy is a simple and effective approach to remove excess tissue iron. For obvious reasons, its use will be limited to patients with good graft function, platelet engfaftment and venous access. Compliance with treatment is usually good and it is a relatively inexpensive procedure to perform. While data in the HSCT population are limited, it has been shown that phlebotomy (alone or with erythropoietin (EPO) support) can effectively reduce serum ferritin levels [64, 76, 79, 80]. A similar program of phlebotomy combined with EPO has been shown to normalize liver enzymes and reduce serum ferritin levels following transplantation [64]. It has been shown that the red cell regeneration induced by EPO is more pronounced in donors with iron overload than in those with normal iron levels [81]. Following HSCT, restoration of normal erythropoiesis allows phlebotomy to be performed and a study in posttransplantation thalassemia patients has shown the potential of this approach [82]. Since many effects of iron overload appear to be mediated by NTBI, it will be important to determine how quickly phlebotomy can normalize NTBI in patients with elevated levels.

### 6.4. Iron Chelation Therapy

**Figure 5:**
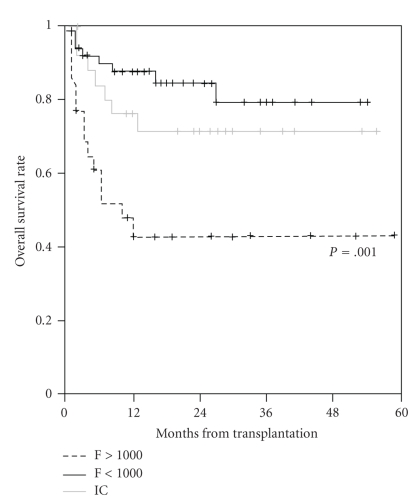
Effect of management of iron levels with iron chelation therapy on outcome in pediatric HSCT patients [30]. Patients receiving iron chelation (IC) therapy to reduce high iron load demonstrated survival levels similar to patients with low serum ferritin (F) levels at transplantation (*N* = 101).

The iron-chelating agent deferoxamine has been available for many years and is the reference standard against which newer therapies are compared. Its use in the HSCT patient is complicated by the very short half life and the ability of deferoxamine to release iron to bacteria and fungi [83, 84]. In one of the earliest studies to evaluate chelation therapy in HSCT, continuous intravenous deferoxamine was administered in two different schedules from day −9 to day +60 after HSCT in patients with thalassemia, effectively reducing serum ferritin levels at 6 months without detrimental effect [85]. In particular, no adverse effects on engraftment or infection risk were noted. Other studies in thalassemia have provided support for the safety and value of chelation therapy started as early as 3 months after HSCT in lowering serum ferritin and hepatic iron [65, 80]. More recently it has been demonstrated in a study of pediatric patients undergoing allogeneic HSCT that pre-transplantation serum ferritin levels of >1000 ng/mL were associated with lower survival. Meanwhile, iron-overloaded patients treated before HSCT with deferoxamine or deferasirox to reduce serum ferritin levels to <1000 ng/mL showed no differences in complications and survival compared with patients whose serum ferritin levels were below 1000 ng/mL before HSCT (Figure 5) [30]. This study provides proof of principle for the value of iron chelation therapy prior to HSCT in order to reduce the complications of HSCT and improve patient outcomes. 

Whilst studies of deferoxamine use in the HSCT setting confirm the value of effective management of iron overload, a key limitation is the demanding regimen of frequent, prolonged infusions (40 mg/kg infused 8–12 hours/day at least 5 days per week). A development in iron chelation therapy came with the availability of the first oral therapy, deferiprone, a thrice-daily formulation that was approved in Europe in 1999 for the treatment of adult patients with thalassemia as second-line therapy when deferoxamine therapy is contraindicated or inadequate. However, this agent has not been investigated in the HSCT setting and is not available in the USA or Canada. Due to the serious nature of agranulocytosis that can occur with the use of deferiprone, close monitoring is required for all patients [86–88]. 

More recently, a once-daily oral therapy, deferasirox, has been licensed in many countries around the world, having been investigated in a wide range of patient types, primarily those with secondary iron overload as a result of chronic red cell transfusions [89–91]. A particular advantage of deferasirox due to its long half-life is its ability to bind LPI on a round-the-clock basis. The ability of deferasirox to normalize LPI by 3 months has been demonstrated in patients with myelodysplasia [92]. Studies are now being conducted in the HSCT setting to determine the efficacy and impact of deferasirox on outcomes in this specific patient group. The common adverse events associated with deferasirox therapy, such as gastrointestinal discomfort and serum creatinine elevation, may, however, overlap with common acute side effects seen after allogeneic transplant, making investigation of deferasirox in early HSCT difficult [54, 93]. In addition, the renal toxicity of deferasirox when used in conjunction with cyclosporine or tacrolimus requires further investigation. Lower doses of deferasirox may be effective in reducing iron burden in patients who have been rendered transfusion-independent by HSCT.

## 7. Conclusions

In recent years, investigation of the association between iron overload and HSCT outcomes has clearly highlighted the adverse impact of elevated serum ferritin levels prior to HSCT on overall survival and complications. While the question of whether a high iron burden contributes directly to poor patient outcome or whether serum ferritin levels act as a surrogate marker for patient prognosis requires further evaluation in prospective multicenter studies, there appears to be no doubt that iron overload can be considered as an independent adverse prognostic factor in allogeneic HSCT, at least in patients with thalassemia, myelodysplasia, and acute leukemia.

The data presented highlight the need to develop effective management strategies in the pre-, peri-, and posttransplantation phases to reduce complications and improve survival. Studies using MRI assessment of tissue iron burden and measurement of LPI at various phases of HSCT and correlating these measures with outcomes will be necessary to fully define the pathophysiology of iron overload in the transplant patient. It is likely that in an individual patient, treatment will require tailoring based on factors such as the nature of the underlying disease, comorbidities, and the distribution of tissue iron.

## Figures and Tables

**Table 1:** Causes of increased NTBI in HSCT recipients.

Source of iron	Underlying mechanism
Increased intestinal iron absorption due to low hepcidin	(i) Feature of some chronic anemias (e.g., MDS, thalassemia intermedia)
	(ii)* HFE* gene mutations

Increased macrophage iron	(i) Red cell transfusion therapy

Under utilization of plasma iron	(i) Inhibition of erythropoiesis as a result of cytotoxic therapy used as part of the conditioning regimen

Release of cellular iron	(i) Destruction of bone marrow and tumor cells as a result of cytotoxic therapy used as part of the conditioning regimen

**Table 2:** Published data showing the impact of pretransplantation serum ferritin levels on survival in HSCT recipients.

	Patient type and number	Type of HSCT	Outcome measures	Key results
Armand et al. 2007 [24]	590 patients with a variety of disorders (primarily CML, AML, or MDS)	Allogeneic (myeloablative)	Retrospective evaluation of 5-year overall survival, treatment-related mortality, disease-free survival and relapse	(i) Elevated pre-transplantation serum ferritin strongly associated with lower overall- and disease-free survival (ii) Subgroup analysis showed association restricted to patients with AML or MDS

Mahindra et al. 2008 [25]	315 Hodgkin or nonHodgkin lymphoma patients	Autologous	Retrospective evaluation of 6-year overall survival and relapse mortality	(i) Pre-transplantation serum ferritin levels of >685 ng/mL associated with significantly lower overall and relapse-free survival (*P* = .002 and .021, resp.)

Pullarkat et al. 2008 [8]	190 patients with lymphoma/myeloma or acute leukemia/myeloid malignancy	Allogeneic (myeloablative)	Prospective evaluation of day 100 survival, acute GVHD and infection complications	Elevated serum ferritin (≥1000 ng/mL) associated with (i) increased mortality and decreased overall survival (*P* = .038 and .004, resp.), (ii) increased acute GVHD and increased blood stream infections (*P* = .009 and .042, resp.)

Platzbecker et al. 2008 [26]	172 patients with MDS	Allogeneic (myeloablative)	Retrospective assessment of the impact of transfusion dependence on patient prognosis	Transfusion dependence did not impact directly on overall survival, but transfusion burden, reflected by serum ferritin, correlated with (i) greater probability of acute GVHD (*P* = .03) (ii) inferior overall survival in patients with serum ferritin levels of >1000 ng/mL (*P* = .03)

Kim et al. 2009 [27]	38 patients with hematologic malignancies	Allogeneic (reduced intensity conditioning)	Retrospective assessment of transplantation outcome after RIST	Elevated serum ferritin (≥1000 ng/mL) resulted in (i) reduced disease-free survival (35.8% versus 80.6% in nonoverloaded patients, *P* = .01); (ii) reduced overall survival (27% versus 54.6% in the iron nonoverload group, *P* = .03)

Kataoka et al. 2009 [28]	264 patients with a variety of disorders, (primarily acute myelogenous/lymphoblastic leukemia, CML or MDS)	Allogeneic (myeloablative and nonmyeloablative)	Retrospective evaluation of 5-year survival, nonrelapse mortality, GVHD and infection	Serum ferritin levels of ≥599 ng/mL resulted in: (i) lower overall survival, higher nonrelapse mortality (*P* < .001) (ii) patients with high serum ferritin levels were more likely to die of infection and organ failure (*P* < .01 and <.019, resp.); (iii) no significant difference in incidence of acute GVHD

Mahindra et al. 2009 [29]	64 patients with a variety of disorders (primarily AML, nonHodgkin lymphoma, or MDS)	Allogeneic (nonmyeloablative)	Prospective evaluation of 5-year survival	(i) Pre-transplantation serum ferritin levels of >1615 ng/mL associated with significantly lower overall survival (*P* = .012)

Lee et al. 2009 [30]	101 pediatric patients with a variety of disorders (primarily acute lymphoblastic/myeloid leukemia, and aplastic anemia)	Allogeneic (myeloablative)	Retrospective analysis of 5-year survival	(i) Serum ferritin levels of >1000 ng/mL associated with reduced overall and event-free survival (*P* = .001)

Mahindra et al. 2009 [31]	222 patients with myeloid or lymphoid leukemia, nonHodgkin lymphoma or MDS	Allogeneic (myeloablative)	Retrospective evaluation of survival and GVHD	Pre-transplantation serum ferritin levels of >1910 ng/mL resulted in (i) reduced overall and relapse-free survival (*P* = .003 and .003, resp.); (ii) reduced chronic GVHD (*P* = .019); (iii) increased nonrelapse mortality (*P* = .042)

AML: acute myeloid leukemia; CML: chronic myeloid leukemia; GVHD: graft-versus-host disease.

**Table 3:** Published guidelines for managing iron overload in HSCT recipients.

Source of guidelines	Focus of guidelines	Recommendations for management of iron load
Nagasaki consensus group (2005) [58]	Consensus statement on iron overload in MDS	(i) Candidates for allograft could benefit from management of iron load with chelation therapy

European Group for Blood and Marrow transplantation, the Center for International Blood and Marrow Transplant Research and the American Society for Blood and Marrow transplantation (2006) [59]	Long-term survivors of HSCT	(i) Most long-term survivors will have some degree of iron overload (ii) LIC >7 mg Fe/g dry weight (dw) should be treated with phlebotomy and/or chelation therapy

MDS Foundation's Working Group on Transfusional Iron Overload (2008) [60]	Consensus statement on iron overload in MDS patients	(i) Allograft candidates may benefit from chelation therapy in order to manage body iron levels prior to transplantation in order to avoid iron-related organ dysfunction and transplant-related morbidity and mortality

Canadian consensus group (2008) [61]	Iron overload in MDS	(i) Consider iron chelation in transfusion-dependent patients who are candidates for allogeneic HSCT
